# In Silico Comparison of Two Kirschner Wire Arrangements for Stabilization of Femoral Capital Physeal Fractures †

**DOI:** 10.3390/vetsci12050422

**Published:** 2025-04-29

**Authors:** Logan M. Scheuermann, Daniel D. Lewis, Richard B. Evans

**Affiliations:** 1 Department of Small Animal Clinical Sciences, College of Veterinary Medicine, University of Florida, Gainesville, FL 326608, USA; 2 Clinical and Translational Science Institute, University of Minnesota, Minneapolis, MN 55414, USA

**Keywords:** capital physeal fracture, capital physeal pinning, femoral fracture, dog, computer-assisted surgery

## Abstract

Despite advancements in surgical techniques, including the utilization of intra-operative fluoroscopy and custom surgical guides, intra-articular implant placement during the pinning of capital physeal fractures can occur and may result in catastrophic complications. Our objective in this study was to compare the epiphyseal purchase afforded by a linear and triangular pattern of Kirschner wires for stabilizing femoral capital physeal fractures. Archived pelvic limb computed tomography scans of 16 skeletally immature dogs were obtained, from which virtual femoral models were created. Separate linear and triangular arrangements of three virtual 1.6 mm Kirschner wires were centered on the thickest region of the proximal epiphysis. The length of each Kirschner wire implanted within the proximal femoral epiphysis was measured. Kirschner wire placement in triangular and linear patterns resulted in a mean epiphyseal purchase for each wire of 8.4 mm and 8.0 mm, respectively. The cumulative Kirschner wire purchase was greater with the triangular pattern (*p* = 0.004). The mean difference in the cumulative wire purchase was 1.3 mm. The use of a triangular Kirschner wire pattern resulted in greater implant purchase within the epiphysis compared to the linear pattern. The triangular Kirschner wire pattern may reduce the risk of intra-articular implant placement by more closely centering the wires within the thickest region of the epiphysis.

## Simple Summary

Penetration of the femoral head and placement of wires within the hip joint is a potentially catastrophic complication that can occur during the stabilization of fractures of the femoral head. This study analyzed two different implant patterns, triangular and linear, using computed tomography scans and virtual bone models to compare the bone purchase afforded with each implant arrangement. The use of implants in a triangular pattern for proximal femoral epiphyseal stabilization resulted in a greater purchase within the epiphysis, which may reduce the risk of implant placement within the joint.

## 1. Introduction

Minimally invasive proximal femoral physeal fracture stabilization has recently been described with Kirschner wires being placed percutaneously, or via a limited approach to the subtrochanteric region of the femur [1,2]. Minimally invasive Kirschner wire placement has been purported to reduce iatrogenic soft tissue trauma and decrease the development of post-operative femoral neck resorption, which has been reported in up to 70% of capital physeal fractures stabilized by open reduction and internal fixation [1,2,3]. In a recent study, de Moya and colleagues reported the results of percutaneous pinning of capital physeal fractures in 11 dogs, with post-operative reduction considered to be anatomic in 10 fractures [1]. Despite the utilization of intra-operative fluoroscopy, one Kirschner wire penetrated the femoral capitis articular surface causing substantial coxofemoral pathology, necessitating implant removal and subsequent femoral head and neck excision [1].

Our group performed a proof-of-concept crossover study using archived computed tomographic (CT) scans of femurs from skeletally immature dogs to plan the placement of three linearly aligned Kirschner wires and created custom surgical guides to facilitate appropriate Kirschner wire placement which could be performed in clinical cases via traditional open reduction or optimally via a limited approach to the proximolateral femur [4]. Participants initially placed Kirschner wires free-hand into three-dimensionally (3D) printed femoral bone models and then subsequently placed Kirschner wires using the custom wire guides. Procedures in which the guides were used were faster and fewer fluoroscopic images were acquired during Kirschner wire placement when utilizing the custom guides. Kirschner wire placement was also more accurate when using the guides, with fewer incidents of wires penetrating the femoral capitus. Ten percent of the linearly aligned Kirschner wires; however, still penetrated the femoral head despite utilizing the guides and fluoroscopy [4]. We speculated that placing Kirschner wires in a triangular pattern may reduce the risk of intra-articular implant placement compared to a linear alignment by more tightly centering the Kirschner wires within the thickest region of the epiphysis [5].

The objective of this study was to compare the amount of epiphyseal purchase afforded by linear and triangular arrangements of Kirschner wires for stabilizing femoral capital physeal fractures. Our hypothesis was that Kirschner wires placed in a triangular pattern would result in greater epiphyseal purchase with a lower risk of penetrating the epiphyseal subchondral bone.

## 2. Materials and Methods

The University of Florida Small Animal Hospital picture archiving and communication system (PACS, Merge Healthcare Inc, Milwaukee, WI, USA) archives were reviewed to identify dogs < 12 months old in which a CT scan (Aquilion Prime S computed tomography scanner, Canon Medical Systems USA, Tustin, CA, USA) of both femora performed between 1 January 2020 and 31 August 2023. Digital Imaging and Communications in Medicine (DICOM) bone algorithm volumetric data files were acquired with a slice thickness of 0.5 mm and an overlap of 0.3 mm. DICOM files were imported into an image processing software program (Mimics 26.0, Materialise, Leuven, Belgium). For each individual dog, either the left or right femur was randomly selected by a coin toss to be used for analysis. The proximal femoral epiphysis and the remainder of the respective femur were segmented separately using Mimics’ predefined bone threshold (i.e., 226 to 3071 Hounsfield units) [6]. Standard tessellation language (STL) files of the virtual femora were exported into a biomodelling software program (3-matic 17.0, Materialise).

**Figure 1:**
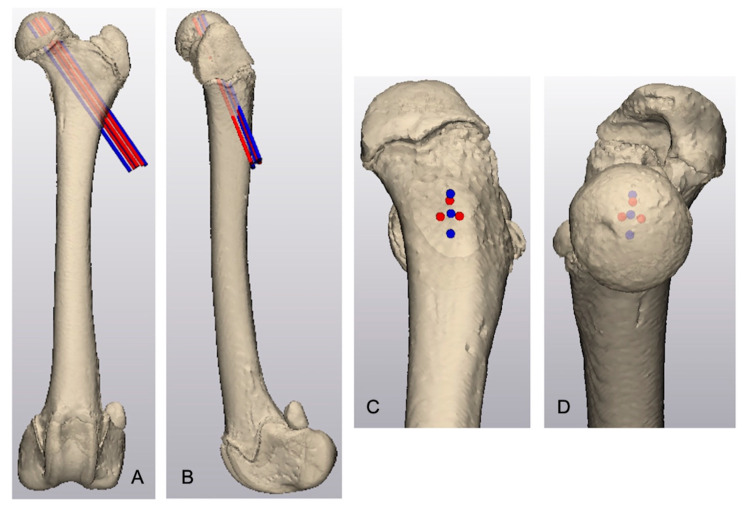
Virtual femoral model with linear (blue cylinders) and triangular (red cylinders) arrangements of virtual 1.6 mm Kirschner wires for pinning of the proximal femoral epiphysis. (**A**) Cranial view, (**B**) lateral view, (**C**) caudolateral view of proximal femur centered on the Kirschner wires, and (**D**) medial view of the proximal femur centered on the Kirschner wires.

The area of maximal thickness of the proximal femoral epiphysis was identified by creating lines oriented perpendicular to the tangent of the subchondral epiphyseal surface and radiated toward the physeal margin of the capitus. The measured length of these lines defined the thickness of the proximal femoral epiphysis. Using the same femur, separate linear and triangular arrangements of three parallel, virtual 1.6 mm cylinders representing Kirschner wires were created. The virtual Kirschner wires in both groups intersected the lateral femoral cortex in the region of the third trochanter and were directed cranioproximally and medially with the proximal aspect of the Kirschner wires centered around the thickest area of the proximal epiphysis (Figure 1). Virtual Kirschner wires were separated by 2 mm and the proximal tip of each Kirschner wire was positioned 2 mm distal to the epiphyseal subchondral bone margin.

Kirschner wires in the linear group were numbered 1 through 3 from proximal to distal. The proximal, craniodistal, and caudodistal Kirschner wires in the triangular group were numbered 1, 2, and 3, respectively. The length of each Kirschner wire seated within the proximal femoral epiphysis was measured. The cumulative epiphyseal purchase for each Kirschner wire in all specimens in each group was calculated by summing the total wire purchase based on wire placement (i.e., Kirschner wire 1, 2, or 3). Wire purchase within the epiphysis was defined as the length of the virtual Kirschner wire that was within the epiphysis. The mean epiphyseal purchase for each Kirschner wire arrangement was calculated as the mean length of wires 1, 2, and 3 within the epiphysis. The difference in the magnitude of Kirschner wire purchase between the arrangements was determined by calculating the difference between the cumulative triangular wire purchase and the cumulative linear wire purchase. An engagement ratio for each Kirschner wire was calculated to determine the percent of epiphyseal purchase for each Kirschner wire [4]. The engagement ratio was calculated by dividing the length of each wire within the proximal epiphysis by the maximal thickness of the epiphysis.

The primary outcome measure was the difference in purchase of the femoral capitus between triangular and linear Kirschner wire arrangements. Given the small sample size of 16 specimens, robust statistical summaries and statistical tests were used to give conservative inferences. Summaries of those differences were reported with medians, 25th percentiles, and 75th percentiles by Kirschner wire placement locations (giving three medians and three sets of percentiles corresponding to placement locations) and for the difference in sums over wire placements, which is interpreted as the difference in the total purchase. Those four medians of differences were tested against zero (i.e., no differences between triangular and linear placements) using Wilcoxon rank-sum tests. Statistical significance was set at *p* < 0.05.

## 3. Results

CT scans of the pelvic limbs of 16 skeletally immature dogs with open capital physes were obtained. The median (range) age of the included dogs was 7 (3–12) months old with a median (range) weight of 23.9 (9.4–52.5) kg. Eleven dogs were intact females, five were intact males, and one was a spayed female. Three dogs were mixed breed dogs and additional breeds included Great Pyrenees (2/16), golden retriever (2/16), American pit bull terrier (2/16), German shepherd (1/16), boxer (1/16), Labrador retriever (1/16), Rhodesian ridgeback (1/16), Newfoundland (1/16), Saint Bernard, and mastiff (1/16).

The mean ± SD maximal epiphyseal thickness was 11.8 ± 2.0 mm (Table 1). The triangular pattern resulted in a mean ± SD epiphyseal purchase of 8.4 ± 1.7 mm for each Kirschner wire while the linear arrangement resulted in a mean ± SD epiphyseal purchase of 8.0 ± 1.7 mm for each wire. Cumulative Kirschner wire purchase was greater for the triangular pattern than the linear pattern in 14 of 16 dogs (*p* = 0.004) with a mean difference in cumulative wire purchase of 1.3 mm per femur. Epiphyseal purchase of Kirschner wires 1 and 3 was greater with the triangular pattern in 63% and 100% of dogs, respectively, with the cumulative difference in purchase for wires 1 and 3 being 2.7 mm and 24.8 mm, respectively. Epiphyseal purchase of Kirschner wire 2 was greater with the linear pattern in 56% of dogs, with a 6.0 mm greater cumulative purchase for Kirschner wire 2 in femurs stabilized with the linear pattern.

## 4. Discussion

The use of a triangular pattern for Kirschner wire placement for the stabilization of the femoral capitus resulted in greater wire purchase of the epiphysis compared to the linear wire pattern. Previous recommendations involved placing the Kirschner wires in the dorsocentral aspect of the femoral capitus to maximize epiphyseal purchase while minimizing the risk of intra-articular implant placement [7,8]. Our results corroborate that the subchondral bone is thickest in the dorsocentral region of the proximal femoral epiphysis and the triangular pattern clusters the Kirschner wires more closely in the dorsocentral region mitigating the risk of intra-articular implant placement.

The complexity of interpreting implant positioning within the spherical femoral head using two-dimensional radiographic imaging can result in unrecognized articular implant penetration despite apparently acceptable implant positioning [8,9]. To emulate optimal wire placement, virtual Kirschner wires were placed 2 mm from the articular margin of epiphyseal subchondral bone to maximize epiphyseal purchase without penetrating the articular cartilage. Recommendations for slipped capital physeal fracture stabilization in human patients suggest seating the tip of pins > 2.5 mm from the margin of the subchondral bone, which has been associated with fewer complications [10]. In a study performed using human cadavers, obtaining maximal purchase of the capitus without the tip of the implant penetrating the articular margin of the subchondral bone can be particularly difficult when performing minimally invasive capital physeal fracture stabilization as intra-operative fluoroscopy was found to overestimate the distance between the tip of the implant and the margin of the subchondral bone [11]. Interpretation of both intra-operative fluoroscopy and conventional radiographs can be challenging. One report suggested a potential 27% artifactual error when using orthogonal imaging to assess appropriate implant placement in the femoral head leading the authors to suggest that implants should be placed within the central two-thirds of the femoral head on orthogonal images to optimize placement [7].

The utilization of the triangular Kirschner wire pattern resulted in a mean increased epiphyseal purchase of 1.3 mm when compared to linear Kirschner wire placement. While centering the Kirschner wires in the thickest aspect of the epiphysis may reduce the risk of penetrating the articular surface, the biomechanical implications resulting from the increased purchase in the proximal epiphysis are unknown. Three virtual Kirschner wires were utilized in the present study as stabilization of simulated capital physeal fractures. Capital physeal fractures stabilized using three Kirschner wires have been shown to be stronger than fractures stabilized with one or two fixation wires [11]. Simulated capital physeal fractures stabilized with three Kirschner wires were also found to be stronger and have a similar stiffness to intact femurs obtained from skeletally immature dogs [12]. In a clinical retrospective study, reviewing the results of femoral slipped capital epiphyseal stabilization in 80 human patients, the probability of having a post-operative complication, including pin penetration of the articular cartilage increased as the number of pins placed increased [10]. Further studies evaluating the biomechanical differences in the triangular and linear wire arrangements may be warranted.

The in silico study design was chosen for the present study as a proof-of-concept to determine if the proposed triangular Kirschner wire pattern would increase the epiphyseal purchase and thus potentially decrease the risk of penetrating the articular surface. However, the virtual design of the study limits the clinical application of the results. Additional studies utilizing cadaveric or simulated bone models are warranted to elucidate if the triangular Kirschner wire pattern reduces the risk of subchondral bone penetration by more tightly centering the wires within the thickest area of the epiphysis. Although the difference in the amount of epiphyseal purchase was greater with the triangular Kirschner wire pattern, a mean increase of 1.3 mm epiphyseal purchase is of unknown clinical relevance. Further studies using 3D-printed femoral models [4], cadaveric studies, and clinical trials are warranted to determine which pattern of Kirschner wire placement may be the most efficacious. Our study design assumed anatomic reduction in the femoral capitus. Non-anatomic reduction in capital physeal fractures has been associated with worse outcomes [7]. If patient-specific guides are used for linear Kirschner wire placement, the risk of intra-articular implant placement may actually be increased if reduction in the femoral capitus is not anatomic as Kirschner wires would likely be advanced into the thinner periphery of the epiphysis. However, more closely clustering the Kirschner wires in a triangular pattern may reduce the risk of intra-articular Kirschner wire placement when stabilizing non-anatomically reduction capital physeal fractures. Further cadaveric and clinical studies are warranted to illustrate which pattern of Kirschner wire placement is more efficacious in maximizing epiphyseal purchase and limiting morbidity due to wires penetrating the articular surface of the femoral head.

## Figures and Tables

**Table 1:** Mean epiphyseal thickness of the femurs and the mean epiphyseal purchase of Kirschner wires in the triangular and linear arrangements. The difference between arrangements was compared and a *p*-value of <0.05 was considered significant.

Wire	Mean Epiphyseal Thickness (mm)	Mean Triangular Wire Purchase (mm)	Mean Linear Wire Purchase (mm)	Wire Purchase Difference (mm)	*p*-Value
1	11.8	9.4	9.1	0.3	0.093
2		8.1	8.4	−0.3	0.193
3		7.7	6.2	1.5	<0.001
Cumulative		8.4	8.0	0.4	0.007

Abbreviations: mm, millimeter.

## Data Availability

The raw data supporting the conclusions of this article will be made available by the authors on request.

## Abbreviations

The following abbreviations are used in this manuscript:

3D	Three-dimensional
CT	Computed tomography
DICOM	Digital imaging and communications in medicine
SD	Standard deviation
STL	Standard tessellation language
